# The SIRT6-Autophagy-Warburg Effect Axis in Papillary Thyroid Cancer

**DOI:** 10.3389/fonc.2020.01265

**Published:** 2020-08-28

**Authors:** Zhou Yang, Renhong Huang, Xiyi Wei, Weiping Yu, Zhijun Min, Min Ye

**Affiliations:** ^1^Department of General Surgery, Shanghai Pudong Hospital, Fudan University Pudong Medical Center, Shanghai, China; ^2^The State Key Lab of Reproductive, Department of Urology, The First Affiliated Hospital of Nanjing Medical University, Nanjing, China

**Keywords:** autophagy, ER stress, reactive oxygen species, SIRT6, warburg effect

## Abstract

As shown in our previous study, SIRT6 promotes an aggressive phenotype and the epithelial-mesenchymal transition (EMT) in papillary thyroid cancer (PTC). In this study, we focused on the regulatory axis including SIRT6, autophagy, and the Warburg effect. We innovatively confirmed that SIRT6 overexpression depleted histone H3 lysine 56 acetylation (H3K56ac) of the negative regulator of reactive oxygen species (NRROS) *in vitro*, thus increasing reactive oxygen species (ROS) production. The accumulated ROS then activated endoplasmic reticulum stress (ER stress) and subsequently induced autophagy. Furthermore, SIRT6 overexpression inhibited glucose transporter 1 (GLUT1) *via* autophagy-mediated degradation, ultimately suppressing the Warburg effect. Treatment with the ROS scavenger N-acetyl-L-cysteine (NAC, 5 mM) or the autophagy inhibitor chloroquine (CQ) both rescued the inhibition of the Warburg effect. Additionally, a higher concentration of NAC (15 mM) further inhibited the Warburg effect. These concentration-dependent bilateral effects of NAC on this process were confirmed to be due to the regulation of the AMPK signaling pathway. Finally, we further examined this mechanism *in vivo* by establishing subcutaneous xenografts in nude mice and analyzed the tumors using 18F radio-labeled fluorodeoxyglucose (18F-FDG) PET/CT. In conclusion, we identified a SIRT6-ROS-ER stress-autophagy-GLUT1-Warburg effect axis in PTC, which may provide a new therapeutic target. In addition, NAC (low concentration) and CQ, previously considered to be tumor inhibitors, were shown to promote tumorigenesis in PTC with high SIRT6 expression by inducing the Warburg effect.

## Introduction

Thyroid cancer is a common malignant tumor, including papillary, follicular, undifferentiated, and medullary pathological types. Among these types, papillary thyroid carcinoma (PTC) is the most common pathological type, accounting for 85% of the total number of cases (1). The incidence of PTC has recently increased annually (2). In the past decade, advances in early detection have improved the prognosis of patients with PTC, but some patients with thyroid cancer still experience a lower benefit and a lower survival rate. Therefore, new targets and new diagnostic markers for PTC are urgently needed (3).

The Sirtuin family, histone deacetylases that depend on nicotinamide adenine dinucleotide (NAD+), consists of seven members (SIRT1-7), each with a different location and function in tissues and cells. SIRT6 is a nuclear histone deacetylase specific for histone H3 lysine 56 acetylation (H3K56ac) and H3K9ac (4, 5). Our previous study had confirmed that SIRT6 increases tumor invasion by inducing the epithelial-mesenchymal transition (6). SIRT6 is also associated with autophagy in bronchial epithelial cells and the Warburg effect in cancer cells (7, 8). However, few studies have focused on SIRT6-induced autophagy in cancer, particularly in thyroid cancer.

Malignant tumors exhibit not only genetic changes but also energy metabolism disorder. Even if a sufficient oxygen supply is available in the microenvironment, tumor cells mainly obtain energy through glycolysis, which is regarded as aerobic glycolysis or the Warburg effect. This abnormal pathway of glucose metabolism promotes glucose uptake and lactate production, which is instrumental in the initiation and development of tumors. The Warburg effect is regulated by a complex network that involves transcription factors, glycolysis enzymes, the PI3K/AKT pathway, reactive oxygen species (ROS), and other factors (9).

Autophagy is a process by which cytoplasmic proteins or organelles are engulfed in vesicles that fuse with lysosomes to form autophagic lysosomes, which degrade the contents to meet the cells' metabolism demands and recycles some organelles. Most malignant tumors exhibit abnormal regulation of the autophagy pathway during many stages of initiation, development, and dissemination. Previous studies of autophagy in thyroid cancer have mainly focused on cell apoptosis and chemosensitivity (10, 11). Autophagy exerts more complex effects on tumors. Based on accumulating evidence, autophagy interacts with the Warburg effect. In cervical cancer, the Warburg effect activates HIF1-α to induce autophagy (12). In colorectal cancer, oridonin induces autophagy by inhibiting the Warburg effect (13). In ovarian cancer, quercetin induces protective autophagy, and apoptosis by activating ER stress through the p-STAT3/BCL-2 axis (14). The specific mechanism underlying the interaction of autophagy and the Warburg effect in PTC remains unclear, therefore, we focused on the regulatory axis of the SIRT6-autophagy-Warburg effect.

## Materials and Methods

### Cell Culture and Regents

TPC-1 and K1 cells (human PTC cell lines) were obtained from the University of Colorado Cancer Center Cell Bank. All cells were cultured in RPMI 1640 medium supplemented with 10% FBS (Invitrogen, Carlsbad, CA, USA) at 37°C in a 5% CO_2_ atmosphere. All experiments were performed with mycoplasma-free cells.

The ROS scavenger N-acetyl-L-cysteine (NAC), histone deacetylase (HDAC) inhibitor trichostatin A (TSA), AMPK pathway activator AICAR, autophagy inhibitor chloroquine (CQ), ER stress inhibitor 4-phenylbutyrate (4-PBA), and proteasome inhibitor MG-132 were all purchased from Cell Signaling Technology. The working concentration and time were confirmed by the manufacturer's instructions combined with the experimental requirements.

### Construction of Stable SIRT6-Overexpressing PTC Cell Lines

The human SIRT6 cDNA was obtained from Origene (RC202833, Rockville, MD, USA) and transferred into the pCDH-CMV-MCS-EF1-Puro lentiviral vector to generate the pCDH-SIRT6 overexpression plasmid. According to the guidance of the product manual, the target plasmid or the empty vector, psPAX2 and PMG.2G, were co-transfected into the HEK293T cells to generate a SIRT6-overexpressing lentivirus or negative control lentivirus with Lipofectamine 3000 (Invitrogen, Inc.). The PTC cell line was then infected with the lentivirus (multiplicity of infection, MOI = 10). Puromycin (1 μg/ml, 72 h) was added to cells to identify the positive clones, and Western blotting and RT-qPCR were used to confirm the overexpression of SIRT6.

### Western Blot Analysis

The total cellular proteins from each group were extracted using RIPA lysis buffer supplemented with 1% phenylmethanesulfonylfluoride fluoride (PMSF). Then, 10% SDS-PAGE gels were utilized to separate equal amounts of protein (20 μg), as determined using the BCA protein assay kit (Thermo Fisher Scientific, USA). Next, the proteins were transferred to PVDF membranes (0.45 mm, Solarbio, China). The membranes were blocked with 5% non-fat milk at room temperature for 1 h and then incubated with the following primary antibodies at 4°C for 12 h: SIRT6, ATF4, CHOP (1:1,000, Proteintech, USA), LC3B, ATG5, Beclin1, GLUT1, NOX2, NRROS (1:1,000, Abcam, USA), p-AKT, AKT, p-mTOR, mTOR, p-ERK1/2, ERK1/2, p-AMPK, and p-eIF2α (1:1,000, Cell Signaling Technology, USA) rabbit polyclonal antibodies. The rabbit polyclonal antibody against β-actin (1:4,000, Proteintech, USA) was used as the loading control and for normalization. The secondary antibodies were anti-mouse or anti-rabbit antibodies conjugated to horseradish peroxidase (HRP) (1:4,000, Proteintech, USA), and were incubated with the membrane at room temperature for ~1 h. The bands were visualized using ECL reagents (Thermo Fisher Scientific, USA) developed by Omega Lum G (Aplegen, USA).

### RNA Extraction, Reverse Transcription, and Quantitative PCR (RT-qPCR)

Total cellular RNA was extracted from PTC cells using Trizol reagent (Invitrogen). Total RNA was reverse transcribed into cDNAs with the PrimeScript™ RT reagent kit (Takara Bio, Inc., Otsu, Japan). Real-time quantitative PCR was utilized to assess SIRT6 expression in triplicate with a SYBR Premix Ex Taq™ kit (Takara Bio) and ABI 7900HT Real-Time PCR system (Applied Biosystems Life Technologies, Foster City, CA, USA). The primers used in this study are listed in Table 1. The final results were analyzed by calculating the comparative cycle threshold values (2^−ΔΔCt^).

**Table 1:** The Primers of RT-qPCR.

**Gene**	**Forward Primer**	**Reverse Primer**
SIRT6	GCACCGTGGCTAAGGCAAGG	GTGATGGACAGGTCGGCGTTC
Beclin1	CAAGATCCTGGACCGTGTCA	TGGCACTTTCTGTGGACATCA
Bnip3	TCCACTTCAGACACCCTA	CTCAGTCGCTTTCCAATA
Vsp34	GGACCTTCTGACCACGAT	GCAACAGCATAACGCCTC
ATG7	TGTATAACACCAACACACTCGA	GGCAGGATAGCAAAACCAATAG
ATG4B	AGAGCCCGTTTGGATACT	GTCGATGAATGCGTTGAG
Actin	GGGACCTGACTGACTACCTC	TCATACTCCTGCTTGCTGAT
PKM	ACTGGCATCATCTGTACCATTG	AGCCACATTCATTCCAGACTTA
LDHA	GGTTGGTGCTGTTGGCATGG	TGCCCCAGCCGTGATAATGA
HK2	CGACAGCATCATTGTTAAGGAG	GCAGGAAAGACACATCACATTT
Glut1	CTGGCATCAACGCTGTCTTC	GCCTATGAGGTGCAGGGTC
Eno1	GCCTCCTGCTCAAAGTCAAC	AACGATGAGACACCATGACG
PGK1	TTCTGTTCTTGAAGGACTGTGT	CTTTAACCTTGTTCCCAGAAGC
GAPDH	GTCAAGGCTGAGAACGGGAA	AAATGAGCCCCAGCCTTCTC
NRROS	AGTCTGCAAGTTGGTGGGTG	GGTCTTGAGAGGGTTGGCAT

### Immunofluorescence (IF) Staining

Regarding cellular Immunofluorescence (IF) staining, after clearing, disinfection, and 24 h of ultraviolet irradiation, coverslips were placed flat on the bottom of a 6-well plate. The cells were then inoculated on the coverslips at a density of 1 × 10^6^ and cultured in an incubator. The coverslips were rinsed with PBS 3 times for 5 min each, fixed with 4% paraformaldehyde for 15 min, and permeabilized with 0.3% TritonX-100 in PBS for 20 min. Next, the coverslips were rinsed again with PBS three times for 5 min each and incubated with 5% BSA for 60 min. Afterwards, the cells were stained with an LC3 (1:100, Abcam, USA) rabbit polyclonal antibody and then incubated at 4°C for 12 h. After the unconjugated antibody was removed by washing the coverslips with PBS three times for 5 min each, the cells were incubated with an Alexa Fluor 498-conjugated goat anti-rabbit IgG secondary antibody (1:200, ProteinTech Group) for 1 h at room temperature in the dark. Finally, DAPI was applied as a counterstaining agent to label the nuclei. Then, the stained cells were photographed with a laser confocal microscope.

For tissue IF staining, tissues were cut into paraffin sections, deparaffinized, and blocked as described in the IHC section. The SIRT6 rabbit antibody was incubated with the sections overnight at 4°C, followed by sequential incubations with the HRP-conjugated rabbit secondary antibody for 1 h and Cy5-conjugated goat anti-HRP antibody (1:200; ProteinTech Group) for 10 min at room temperature. Sections were then boiled in 1 mM EDTA and then heated for 15 min at a sub-boiling temperature to remove the antibodies that have been incorporated into tissues. Similar to SIRT6 staining, sections were incubated with antibodies against GLUT1 and P62, followed by HRP-conjugated rabbit/mouse secondary antibodies and a Cy3-conjugated goat anti-HRP antibody and FITC-conjugated goat (1:200; ProteinTech Group) anti-HRP antibody (1:200; ProteinTech Group), respectively. In the same experiment, DAPI was applied as a counterstaining agent to label the nuclei. The stained sections were then collected and photographed using a fluorescence microscope.

### Chromatin Immunoprecipitation (ChIP) Assay

Chromatin immunoprecipitation (ChIP) was performed using the Magna ChIP Kit (Millipore, Darmstadt, Germany) according to the manufacturer's instructions. Normal mouse IgG as a negative control, or H3K56ac (Active motif, USA), or SIRT6 (Abcam, USA) antibodies were applied to immunoprecipitate chromatin samples. Subsequently, IP was performed with RT-qPCR as described above. The primers for the NRROS promoter were forward: 5'-GGACTTGGCTCCTGTTCTCTT-3' and reverse: 5'-TGACGTGGACCACCATAGTC-3'.

### Transmission Electron Microscopy (TEM)

First, 4 × 10^6^ cells were harvested and fixed with 2.5% glutaraldehyde/PBS for 2 h at 4°C. The cell pellet was embedded in 1% agarose and washed with 0.1 M PBS three times for 15 min each. The samples were further fixed with 1% OsO_4_ in PBS (pH 7.4) for 2 h, washed three times with PBS, and then dehydrated with a series of ethanol solutions (50, 70, 80, 90, 95, and 100%) at an interval of 15 min. The samples were subsequently incubated with a mixture of alcohol and isoamyl acetate for 30 min, followed by an incubation with pure isoamyl acetate for 1 h. Finally, the samples were coated with gold-palladium, cut into ultrathin sections, and observed with a transmission electron microscope (TEM; Hitachi HT7700, Tokyo, Japan).

### Glucose Uptake, Lactate Production, and ATP Content Assays

For the assays of glucose uptake and lactate production, 5 × 10^5^ cells were inoculated onto 6-well culture plates and incubated at 37°C for 24 h. Afterwards, 5 μl of the cell culture supernatant were collected in 96-well plates and mixed with 200 μl of the Glucose (HK) Assay Reagent (Sigma-Aldrich, CA, USA) or 100 μl of Lactate Assay Regent (Sigma-Aldrich, CA, USA). After an incubation for 20 min at 37°C, a microplate reader was used to measure the absorbance at 340 nm (OD_340_). Meanwhile, a standard curve was generated with glucose and lactate standards. Glucose or lactate levels were obtained by substituting the OD_340_ of the sample into a standard curve. For the assessment of the ATP content, 2 × 10^3^ cells were inoculated onto 96-well plates and cultured for 48 h. After removing the culture medium, the ATPLite luminescent assay (PerkinElmer, Inc., Waltham, MA, USA) reagent was added. After an incubation at 37°C for 30 min, luminescence was observed. Meanwhile, a standard curve was constructed with an ATP standard. ATP levels were obtained by substituting the luminescence intensity into the standard curve. The cell number was used to normalize the glucose uptake, lactate production, and ATP content.

### Measurement of the Extracellular Acidification Rate and Oxygen Consumption Rate

The extracellular acidification rate (ECAR) and cellular oxygen consumption rate (OCR) of cells were measured using the Seahorse XFe 96 Extracellular Flux Analyzer (Seahorse Bioscience) with the Glycolysis Stress Test Kit and Cell Mito Stress Test Kit, respectively (Agilent Technologies). Both tests were performed according to the manufacturer's instructions. Briefly, 10^4^ cells were inoculated into each well of a Seahorse XF 96-well cell culture microplate, incubated for 24 h, and treated with CQ or NAC as described above. For the ECAR measurement, glucose (10 mM), the oxidative phosphorylation inhibitor oligomycin (1 μM), and the glycolysis inhibitor 2-DG (50 mM) were sequentially injected into each well at the indicated time points. For the OCR assay, oligomycin (1 μM), the reversible inhibitor of oxidative phosphorylation FCCP (p-trifluoromethoxy carbonyl cyanide phenylhydrazone, 1 μM), and the mitochondrial complex I inhibitor rotenone plus the mitochondrial complex III inhibitor antimycin A (Rote/AA, 1 μM) were sequentially injected. The protein concentration determined with the BCA assay was used to normalize all data as described above.

### ROS Detection and Inhibition

For ROS detection, 5 × 10^5^ cells were inoculated onto 6-well culture plates and incubated at 37°C for 24 h. After removing the culture medium, 1 ml of FBS-free medium containing diluted 2,7-dichlorodihydrofluorescein diacetate (DCFH-DA; Beyotime, Shanghai, China) was added to the dish. After an incubation at 37°C for 20 min, cells were washed with FBS-free medium three times. A microplate reader was then used to detect the fluorescence intensity of the cells. Notably, 5 mM NAC was added to the medium for 3 h to inhibit ROS, and the inhibitory effect on ROS was confirmed using the DCFH-DA method.

### Establishment of Subcutaneous Xenografts in Nude Mice

Five-week-old male Balb/c-nu mice were obtained from the Beijing Vital River Laboratory Animal Technology Co. Ltd. All detailed experimental procedures were approved by the Institutional Animal Care and Utilization Committee of Fudan University Pudong Animal Experimental Center. All mice (*n* = 30) were equally and randomly separated into the TPC1-NC, TPC1-SIRT6, TPC1-SIRT6+CQ, TPC1-SIRT6+NAC (low), and TPC1-SIRT6+NAC (high) groups. Next, 2 × 10^7^ TPC1-NC or TPC1-SIRT6 cells suspended in 100 μl of PBS were subcutaneously injected into the axilla of each nude mouse. After 2 weeks, NAC (low: 50 mg/kg; high: 150 mg/kg) dissolved in 100 μl of normal saline was intraperitoneally injected into the TPC1-SIRT6 + NAC group (once every 2 days for 21 days). CQ (50 mg/kg) was injected under the same conditions into the TPC1-SIRT6+CQ group, and the TPC1-NC and TPC1-SIRT6 groups were treated with 100 μl of normal saline as a placebo. Vernier calipers were used to measure the long (L) and short (S) diameters of the tumors every 3 days (tumor volume = L^*^S^2^/2). The growth curve of subcutaneous tumors was drawn based on the measured tumor volume. All mice were euthanized after 2 weeks of treatment and subcutaneous tumors were completely removed.

### PET Imaging of Glucose Uptake in Mice

Animal PET scanners (Siemens Corp.) were used for PET imaging of mice. After anaesthetization with pentobarbital, mice were intravenously injected with 3.7 MBq (100 mCi) of 18F radio-labeled fluorodeoxyglucose (18F-FDG). Five-minute emission scans were recorded to obtain attenuation correction data in the prone position at 60 min after the injection, and 10-min delayed scans were acquired at 2 h.

### Patients and Specimens

We collected PTC specimens between July 2018 and July 2019. Patients who met the following criteria were excluded: received adjuvant chemotherapy or radiotherapy before surgery and diagnosed with other cancers. All patients were categorized according to the 7th edition of the TNM staging system 23. All patients received relevant adjuvant treatment according to the standard treatment strategy. All enrolled patients signed a written informed consent form. This study was approved by the Ethics Committee of Shanghai Pudong Hospital.

### Statistical Analysis

All experiments were conducted at least three times. Statistical analyses were performed on all experimental data with SPSS software (version 19.0, IBM Corp., Armonk, NY, USA). Statistical results were determined using GraphPad Prism (version 7, GraphPad Software, La Jolla, California, USA). All data are presented as means + standard deviations (means + sd). Statistical analyses of two sets of data were performed using the *t*-test. Comparisons of data from multiple groups were conducted using one-way analysis of variance followed by the LSD *t*-test. *P* < 0.05 was regarded as significant.

## Results

### SIRT6 Induced Autophagy in PTC

**Figure 1:**
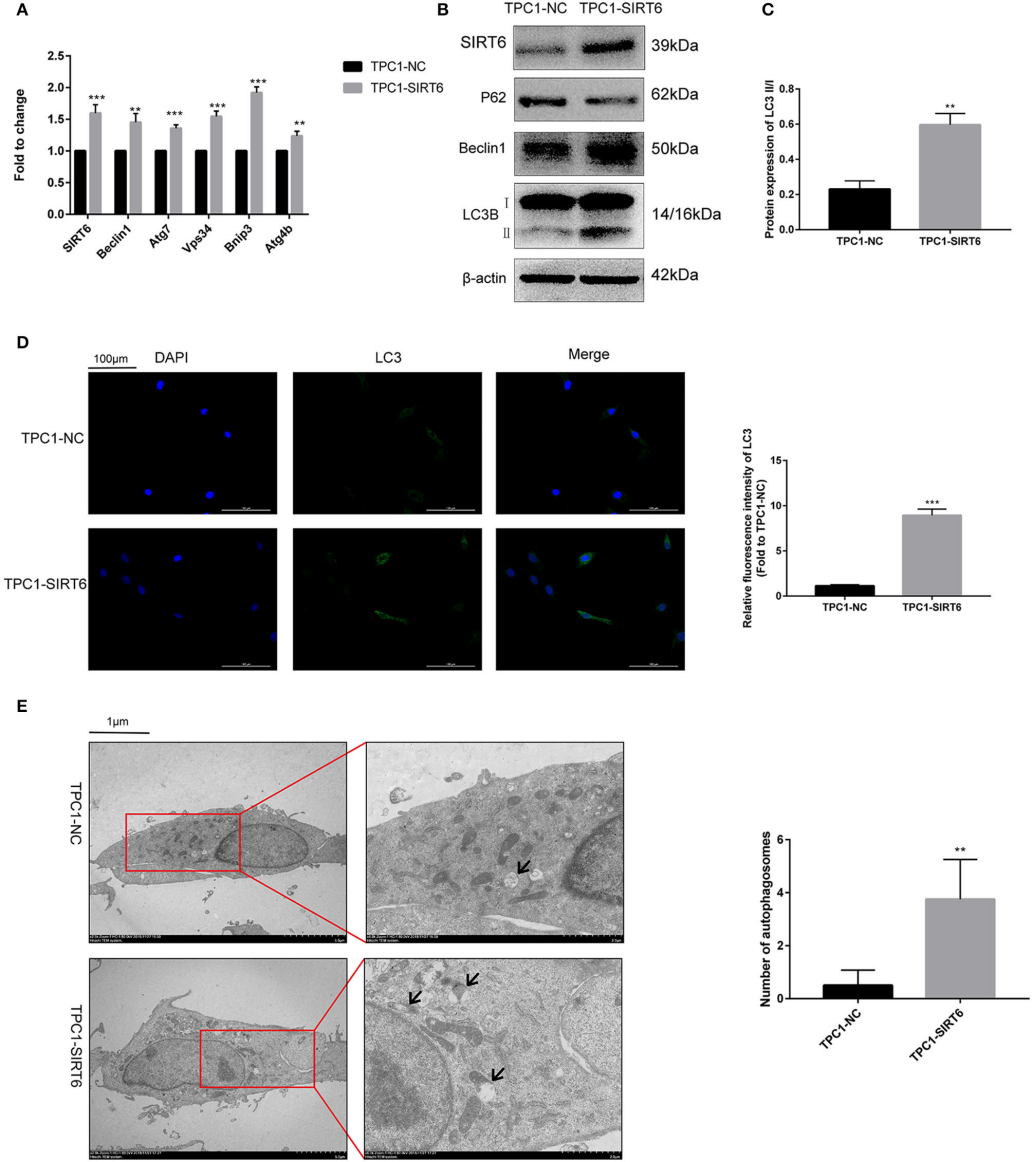
SIRT6 induced autophagy in PTC cells. **(A)** The mRNA expression of autophagy-associated genes (Beclin1, ATG7, VPS34, BNIP3, and ATG4B) in TPC1-SIRT6 and TPC1-NC cells was detected using RT-qPCR. **(B,C)** Levels of autophagy-associated proteins (P62, Beclin1, and LC3B) in TPC1-SIRT6 and TPC1-NC cells were detected using Western blotting. **(D)** Images of immunofluorescence staining for LC3 in TPC1-SIRT6 and TPC1-NC cells. **(E)** Autophagosomes observed using TEM in TPC1-SIRT6 and TPC1-NC cells (arrow: autophagosome). (**p < 0.01 and ***p < 0.001).

The PTC cell line TPC1-SIRT6 stably overexpressing SIRT6 was successfully constructed and further confirmed using Western blotting and RT-qPCR. Compared with TPC1-NC cells, the expression of the SIRT6 mRNA (Figure 1A) and protein (Figures 1B,C) was significantly increased in TPC1-SIRT6 cells. Moreover, the mRNA expression of the autophagy-associated genes Beclin1, ATG7, VPS34, BNIP3, and ATG4B was significantly upregulated in TPC1-SIRT6 cells compared with TPC1-NC cells. Similarly, in the Western blot analysis, the LC3II/I ratio and levels of Beclin1 and ATG5 were significantly increased, whereas P62 levels were significantly decreased in TPC1-SIRT6 cells compared with TPC1-NC cells. In addition, TPC1-SIRT6 cells showed a stronger fluorescence intensity for LC3 than TPC1-NC cells (Figure 1D), and TPC1-SIRT6 cells showed a lower fluorescence intensity for P62 than TPC1-NC cells (Figure S1I). Finally, TEM was performed to directly observe autophagosomes (Figure 1E). We successfully observed more autophagosomes in TPC1-SIRT6 cells than in TPC1-NC cells. Based on these results, SIRT6 induces autophagy in TPC-1 cells, and similar results were obtained in the K1 cell line (Figures S1B,H).

### SIRT6 Promoted Autophagy via ROS-Induced ER Stress

**Figure 2:**
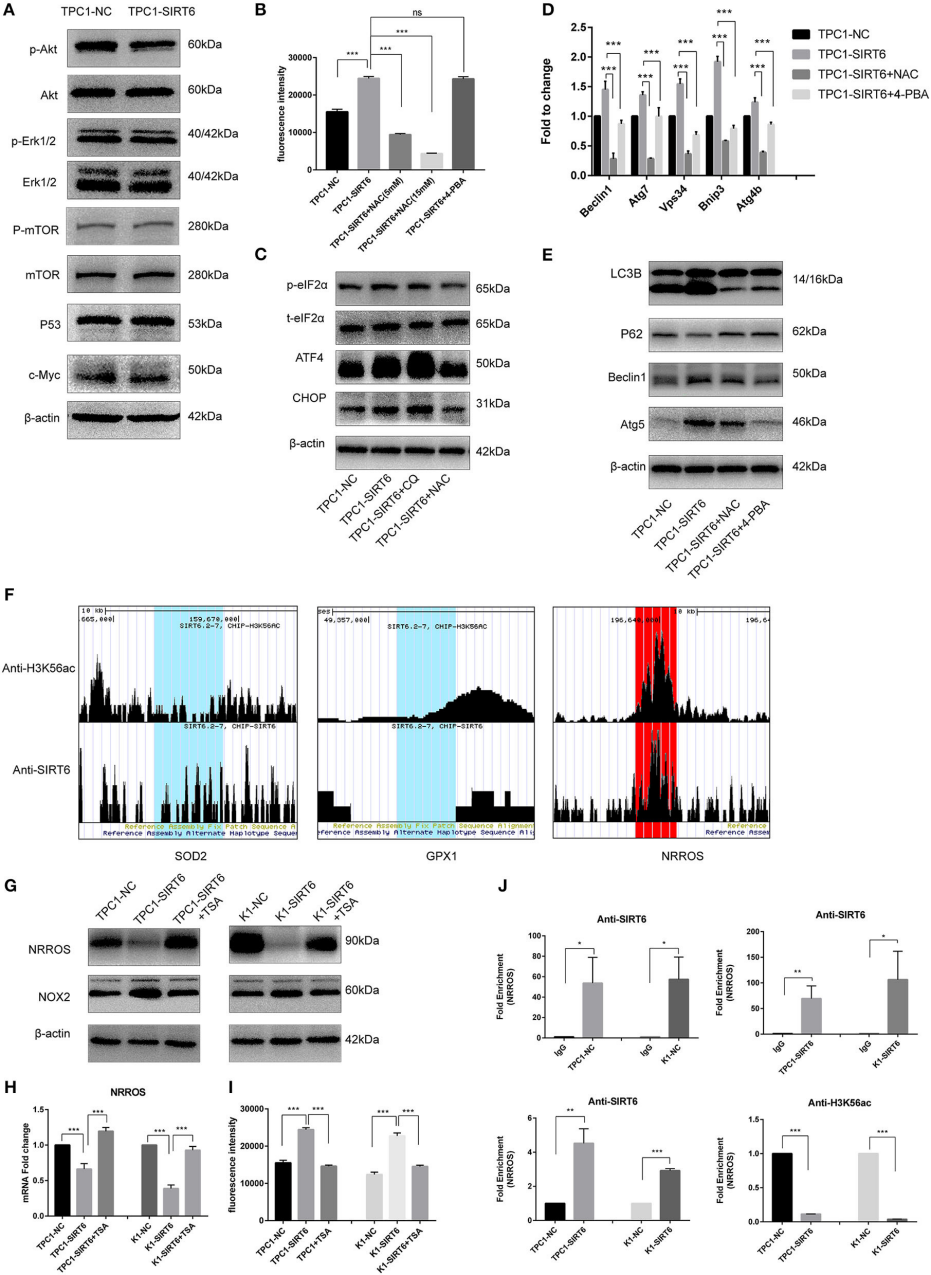
SIRT6 promoted autophagy *via* ROS-induced ER stress. **(A)** Levels of proteins involved in common autophagy-associated pathways (AKT/mTOR and ERK/mTOR) and transcription factors (P53 and c-Myc) in TPC1-SIRT6 and TPC1-NC cells. **(B)** ROS production in TPC1-NC, TPC1-SIRT6, and TPC1-SIRT6 cells treated with CQ (50 μM for 3 h) and the ER stress inhibitor 4-PBA (2 mM for 24 h) was detected using DCFH-DA. **(C)** Levels of proteins involved in the ER stress-associated PERK/ATF4/CHOP pathway in TPC1-NC, TPC1-SIRT6, and TPC1-SIRT6 cells treated with CQ and the ROS inhibitor NAC (5 mM for 3 h). **(D)** The mRNA expression of autophagy-associated genes (Beclin1, ATG7, VPS34, BNIP3, and ATG4b) in TPC1-NC, TPC1-SIRT6, and TPC1-SIRT6 cells treated with CQ and 4-PBA. **(E)** Levels of autophagy-associated proteins (LC3B, P62, ATG5, and Beclin1) in TPC1-NC, TPC1-SIRT6, and TPC1-SIRT6 cells treated with CQ and NAC. **(F)** ChIP-sequencing results obtained from SIRT6-depleted BRAF^V600E^ melanoma cells extracted from GSE102813. **(G)** Levels of the NRROS and NOX2 proteins in TPC1-NC, TPC1-SIRT6, and TPC1-SIRT6 cells treated with the histone deacetylation inhibitor TSA (2 mM for 24 h). **(H)** Expression of the NRROS mRNA in TPC1-NC, TPC1-SIRT6, and TPC1-SIRT6 cells treated with TSA. **(I)** ROS production in TPC1-NC, TPC1-SIRT6, and TPC1-SIRT6 cells treated with TSA. **(J)** Enrichment of SIRT6 and H3K56ac at the NRROS promoter in PTC cells (TPC1-NC, TPC1-SIRT6, K1-NC, and K1-SIRT6) detected using the ChIP analysis. (*p < 0.05, **p < 0.01, and ***p < 0.001).

Western blotting was performed to detect proteins involved in two of the most common pathways of autophagy, AKT/mTOR, and ERK/mTOR, and to explore the mechanism by which SIRT6 induced autophagy (15). Although p-Akt levels were reduced in TPC1-SIRT6 cells, a significant alteration in the levels of its downstream protein p-mTOR was not observed. The ERK/mTOR signaling pathway was not activated. Moreover, two of the most common transcriptional factors associated with autophagy, P53 and c-Myc, were detected, and no changes were observed (Figure 2A). ROS-induced ER stress promotes autophagy, and thus, we further detected ROS levels in cells using 2,7-dichlorodihydrofluorescein diacetate (DCFH-DA) (16). A significant increase in ROS levels was observed in TPC1-SIRT6 cells compared with TPC1-NC cells. The increase in ROS levels observed in TPC1-SIRT6 cells was inhibited by the ROS inhibitor NAC (5 or 15 mM for 3 h), whereas the ER stress inhibitor 4-PBA (2 mM for 24 h) did not alter ROS levels (Figure 2B). Additionally, the ER stress-associated p-eIF2α/ATF4/CHOP pathway was activated in TPC1-SIRT6 cells compared with TPC1-NC cells. NAC (5 mM for 3 h), but not CQ (50 μM for 3 h), successfully inhibited p-eIF2α/ATF4/CHOP signaling (Figure 2C). Therefore, we postulated that SIRT6 induced ER stress by increasing ROS production, and autophagy may be its downstream reaction. TPC1-SIRT6 cells were treated with NAC and 4-PBA to detect the expression of autophagy-associated genes and to further examine the association of SIRT6-induced ER stress and autophagy. Both NAC (5 mM) and 4-PBA successfully rescued the mRNA expression of autophagy-associated genes in TPC1-SIRT6 cells (Figure 2D). Similar results were obtained in the Western blot analyses, as the ratio of LC3II/I and levels of Beclin1, ATG5, and P62 were rescued by the NAC and 4-PBA treatments (Figure 2E). Similar results were also obtained in the K1 cell lines (Figures S1A,B,D). Thus, far, we have confirmed that SIRT6 induced ER stress by increasing ROS production and subsequently activating autophagy. However, it was not clear how SIRT6 increased ROS production. SIRT6 is a class III histone deacetylase (HDAC) that inhibits H3K56ac and H3K9ac. We investigated the ChIP-sequence dataset GSE102813, which studied SIRT6 depletion in BRAF^V600E^ melanoma cells (17). The expression of the main ROS negative regulators was assessed, and SIRT6 bound to the NRROS promoter, accompanied by a significant H3K56ac peak. Negative results were obtained for the other ROS scavengers, such as superoxide dismutase 2 (SOD2) and glutathione peroxidase (GSH-px, GPX1) (Figure 2F). NRROS is a recently reported ROS regulator that inhibits ROS by interacting with NOX2 (18). SIRT6 overexpression significantly repressed the expression of NRROS but induced the expression of NOX2. Moreover, treatment with the HDAC inhibitor trichostatin A (TSA, 400 nM for 12 h) rescued the changes in both NRROS and NOX2 expression (Figure 2G). Furthermore, based on the results of the RT-qPCR analysis, SIRT6 inhibited NRROS at the transcriptional level, and TSA rescued the expression of the NNROS mRNA (Figure 2H). Similar to the changes observed in NRROS expression, TSA successfully rescued the decrease in ROS production in TPC1-SIRT6 and K1-SIRT6 cells (Figure 2I). To research specific mechanisms at the transcriptional level, an A ChIP analysis was performed to investigate specific mechanisms at the transcriptional level. The NRROS promoter region showed significant enrichment in each group with anti-SIRT6 ChIP, and SIRT6 overexpression further promoted the enrichment. In addition, SIRT6 overexpression also depleted H3K56ac at the NRROS promoter (Figure 2J). Thus, SIRT6 binds to the NRROS promoter and depletes H3K56ac, thus suppressing its expression at the transcriptional level. Finally, the inhibition of NRROS negatively regulated ROS levels by altering NOX2 expression.

### SIRT6 Inhibited the Warburg Effect by Inducing the Autophagic Degradation of GLUT1

**Figure 3:**
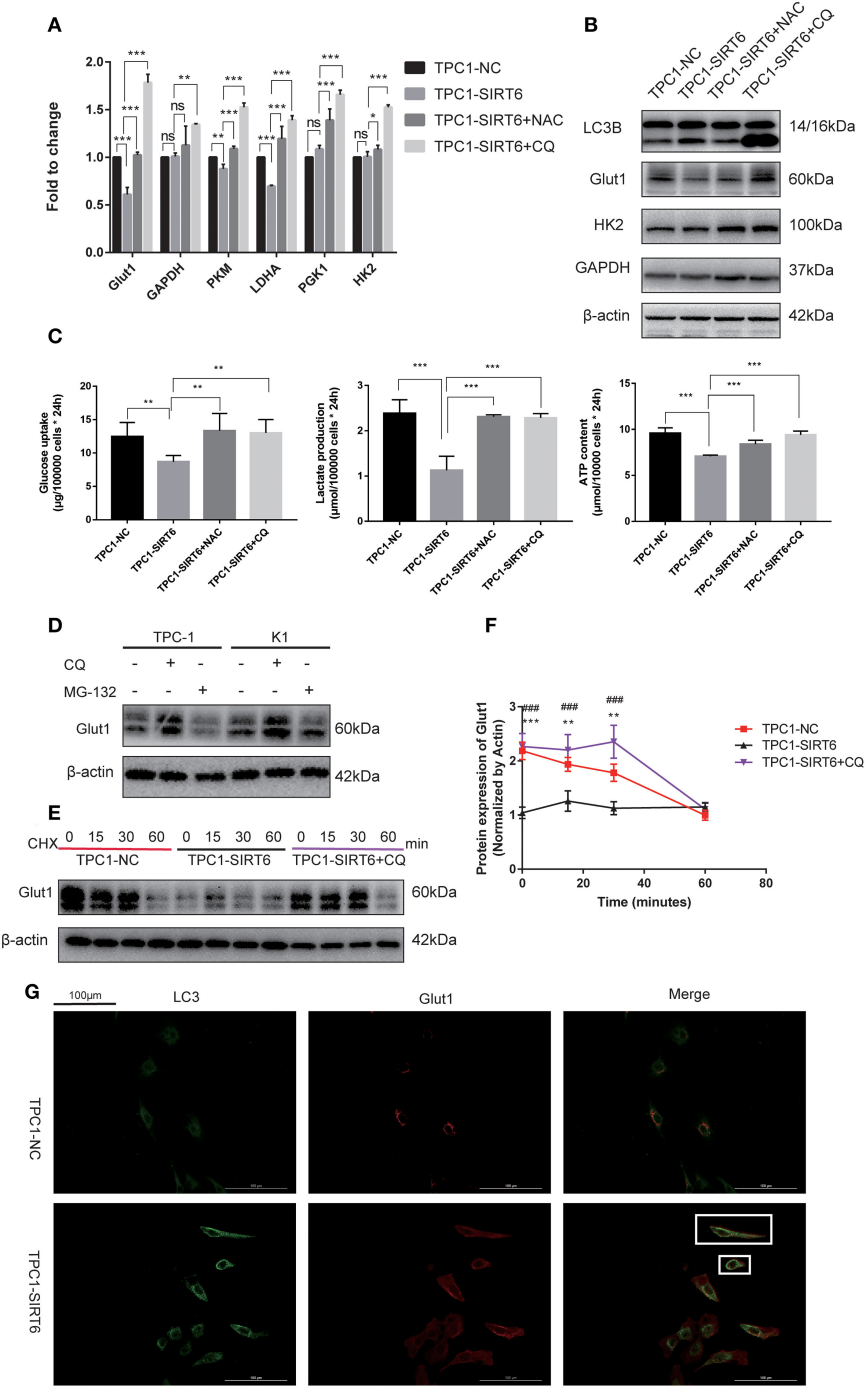
SIRT6 inhibited the Warburg effect by inducing the autophagic degradation of GLUT1. **(A)** The mRNA expression of Warburg effect-associated genes (GLUT1, GAPDH, PKM, LDHA, PGK1, and HK2) in TPC1-NC, TPC1-SIRT6, and TPC1-SIRT6 cells treated with CQ and NAC. **(B)** Levels of proteins involved in the Warburg effect (GLUT1, HK2, and GAPDH) in TPC1-NC, TPC1-SIRT6, and TPC1-SIRT6 cells treated with CQ and NAC. **(C)** Glucose uptake, lactate production and ATP concentrations in TPC1-NC, TPC1-SIRT6, and TPC1-SIRT6 cells treated with CQ and NAC (*p < 0.05, **p < 0.01, and ***p < 0.001). **(D)** Levels of the GLUT1 proteins in TPC-1 and K1 cells treated with CQ and the proteinase inhibitor MG-132 (10 μM for 6 h). **(E,F)** Levels of the GLUT1 protein at different time points (0, 15, 30, and 60 min) after the withdrawal of the 24-h CHX treatment in TPC1-NC, TPC1-SIRT6, and TPC1-SIRT6 cells treated with CQ (*TPC1-NC vs. TPC1-SIRT6, **p < 0.01 and ***p < 0.001; #TPC1-SIRT6 vs. TPC1-SIRT6+CQ, ^###^*p* < 0.001). **(G)** Images of immunofluorescence staining for GLUT1 and LC3 in TPC1-NC and TPC1-SIRT6 cells.

Both RT-qPCR and Western blotting were performed to assess the association of the Warburg effect and autophagy *in vitro*. The mRNA expression of key genes related to the Warburg effect, GLUT1, PKM, and LDHA, was significantly decreased in TPC1-SIRT6 cells compared with TPC1-NC cells, whereas the expression of GAPDH, HK2, and PGK1 showed no changes (Figure 3A). Levels of the GLUT1 protein were significantly decreased in the TPC1-SIRT6 cells, whereas HK2 and GAPDH levels were not altered (Figure 3B). We further examined the glucose uptake and lactate release into the cell culture medium and observed an apparent decrease in these two parameters in the TPC1-SIRT6 cells compared with the TPC1-NC cells. Then, the ATP content was measured using the ATPLite luminescent assay. The same trend of a lower ATP content was detected in the TPC1-SIRT6 cells compared with TPC1-NC cells (Figure 3C). Based on these results, SIRT6 inhibited the Warburg effect *in vitro*. TPC1-SIRT6 cells were treated with the autophagy inhibitor CQ (50 μM for 3 h) or ROS inhibitor NAC (5 mM for 3 h) to further explore the role of ROS in SIRT6-regulated autophagy and the Warburg effect. Both CQ and NAC rescued the mRNA expression of genes involved in the Warburg effect to different extents (Figure 3A). In the Western blot analysis, GLUT1 levels were increased in NAC- and CQ-treated TPC1-SIRT6 cells compared with control TPC1-SIRT6 cells. However, we did not observe noticeable differences in HK2 or GAPDH levels between the TPC1-SIRT6 and TPC1-NC groups. Interestingly, both the NAC and CQ treatments significantly increased GAPDH and HK2 expression compared with the control TPC1-SIRT6 cells (Figure 3B). Furthermore, glucose uptake, lactate production, and ATP concentrations were measured repeatedly, and both NAC and CQ rescued these measurements to different extents (Figure 3C). Similar results were also obtained from K1 cell lines, and these results indicated that SIRT6 inhibited the Warburg effect through ROS-induced autophagy (Figures S1C–G). SIRT6 increased the level of the GLUT1 protein, but not the HK2 and GAPDH proteins. Additionally, GLUT1 levels exhibited the most significant decrease among all factors analyzed using RT-qPCR. Therefore, we considered GLUT1 as the primary factor by which SIRT6 regulated the Warburg effect. The level of the protein is influenced by the level of the transcript, translation and post-translational modifications. The autophagy-lysosomal pathway is an important mechanism that affects the level of protein translation by regulating protein degradation. Therefore, we further investigated the degradation of GLUT1. The lysosome inhibitors CQ (50 μM for 6 h) and protease inhibitor MG-132 (10 μM for 6 h) were used to treat PTC cells. As expected, CQ but not MG-132 significantly increased the expression of GLUT1, indicating that the autophagy-lysosomal pathway may be the main pathway mediating the degradation of GLUT1 (Figure 3D). Subsequently, each group of cells was treated with cycloheximide (CHX), a protein synthesis inhibitor, at a dose of 50 μg/ml for 24 h. CHX was then removed and proteins were extracted at different time points (0, 15, 30, and 60 min) after the withdrawal, to analyze the degradation of GLUT1. GLUT1 was expressed at much lower levels in TPC1-SIRT6 cells than in TPC1-NC cells at each time point (except for 60 min), whereas CQ significantly restored the expression in TPC1-NC cells (Figure 3E). Interestingly, the rate of GLUT1 degradation in TPC1-SIRT6 cells was slower than in TPC1-NC and TPC1-SIRT6+CQ cells (Figure 3F). We hypothesized that the low expression of GLUT1 in TPC1-SIRT6 cells triggers other feedback regulatory mechanisms to antagonize the SIRT6-induced degradation. Subsequently, IF staining was performed to further determine the relationship between GLUT1 degradation and SIRT6-induced autophagy. In TPC1-NC cells, GLUT1 was localized in the membrane and LC3 showed a diffuse and weak distribution in the cytoplasm. However, in TPC1-SIRT6 cells, GLUT1 was localized in the cytoplasm, and exhibited co-localization with the strongly expressed LC3 (Figure 3G). Based on these results, SIRT6 reduced the membrane expression of Glut1 *via* the autophagy-lysosome pathway. In addition, similar results were also obtained in K1 cell lines (Figures S2A,B). Thus, SIRT6 inhibited the Warburg effect *via* ROS-induced autophagy. The autophagic degradation of GLUT1 was responsible for the inhibition of this pathway.

### Greater Inhibition of ROS Production Restrained the Warburg Effect by Suppressing the AMPK Pathway

**Figure 4:**
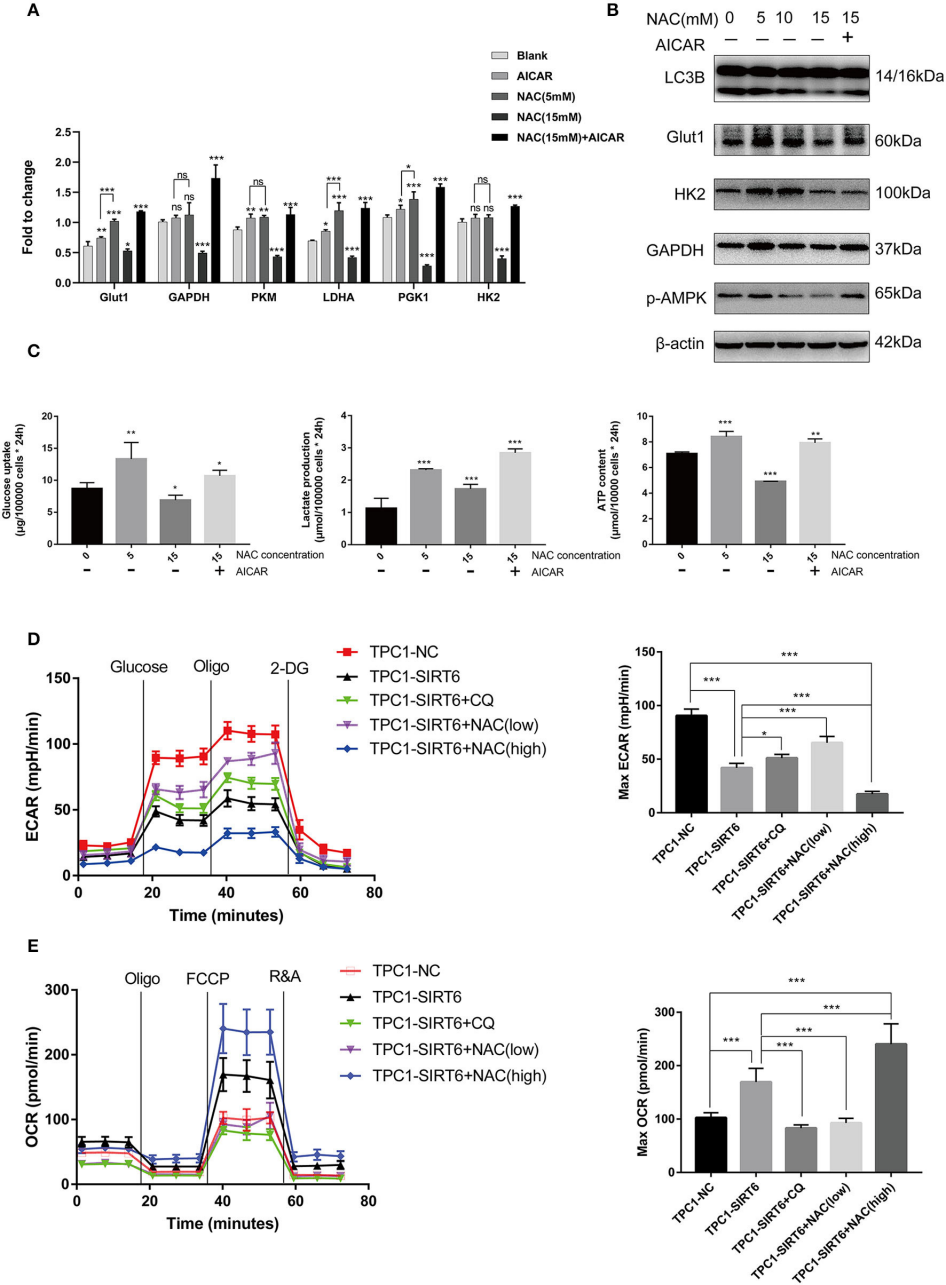
A higher level of inhibition of ROS production restrained the Warburg effect by suppressing the AMPK pathway. **(A)** The mRNA expression of Warburg effect-associated genes (GLUT1, GAPDH, PKM, LDHA, PGK1, and HK2) in TPC1-SIRT6 cells treated with different concentration of NAC (0, 5, or 15 mM for 3 h) and the AMPK pathway activator AICAR (2 mM for 3 h). **(B)** Levels of proteins associated with the Warburg effect (GLUT1, HK2, and GAPDH), LC3B, and p-AMPK in TPC1-SIRT6 cells treated with different concentrations of NAC and the AMPK pathway activator AICAR. **(C)** Glucose uptake, lactate production and ATP concentrations in TPC1-SIRT6 cells treated with different concentrations of NAC and the AMPK pathway activator AICAR. (All compared with TPC1-SIRT6+NAC 0 mM, *p < 0.05, **p < 0.01, and ***p < 0.001). **(D)** ECAR of TPC1-NC, TPC1-SIRT6, and TPC1-SIRT6 cells treated with CQ and NAC (low: 5 mM for 3 h, high: 15 mM for 3 h) detected using the Seahorse XF 96 analyzer. **(E)** OCR of TPC1-NC, TPC1-SIRT6, and TPC1-SIRT6 cells treated with CQ and NAC (low: 5 mM for 3 h, high: 15 mM for 3 h) detected using the Seahorse XF 96 analyzer. (*p < 0.05 and ***p < 0.001).

Increased ROS production has been shown to induce the Warburg effect (19), which contradicts our findings described above. TPC1-SIRT6 cells were treated with the AMPK activator AICAR and a higher concentration of NAC to further explore the mechanism by which ROS regulate the Warburg effect. AICAR also induced the expression of Warburg effect-associated genes, including GLUT1, PKM, LDHA, and PGK1, but to a lesser extent than NAC (5 mM). Interestingly, 15 mM NAC restrained the expression of all Warburg effect-associated genes in TPC1-SIRT6 cells (Figure 4A). This result differed from the effect of 5 mM NAC (which rescued the expression of Warburg effect-associated genes). In the Western blot analysis, an increasing NAC concentration gradually reduced the levels of autophagy-related proteins. The treatment with 15 mM NAC decreased the levels of proteins associated with the Warburg effect, in contrast to the treatment with 5 mM NAC (Figure 4B). Furthermore, 15 mM NAC was sufficient to inhibit glucose uptake and lactate production and decrease the ATP content of TPC1-SIRT6 cells (Figure 4C). In the Western blots, p-AMPK levels did not decrease in cells treated with 5 mM NCA. However, when the concentration of NAC reached 10–15 mM, p-AMPK levels were decreased significantly. Subsequently, TPC1-SIRT6 cells were simultaneously treated with the AMPK activator AICAR (2 mM, 3 h) and NAC (15 mM, 3 h). AICAR successfully activated the AMPK pathway and increased the ratio of LC3II/I. Furthermore, the expression of Warburg effect-associated genes, glucose uptake, lactate production, and the ATP content also rescued to different extents. An increase in the ECAR and decrease in the OCR are the two main indexes reflecting the Warburg effect (20). As expected, overexpression of SIRT6 significantly decreased the ECAR and increased the OCR, indicating that SIRT6 suppressed the Warburg effect. Both CQ and NAC (5 mM) rescued the changes induced by SIRT6 overexpression. In addition, a higher concentration of NAC (15 mM) aggravated the inhibition of the Warburg effect in TPC1-SIRT6 cells (Figures 4D,E). Similar results were also obtained in K1 cells (Figures S2C,D).

### CQ and NAC Promoted the Growth of SIRT6-Overexpressing PTC Cells *in vivo*

**Figure 5:**
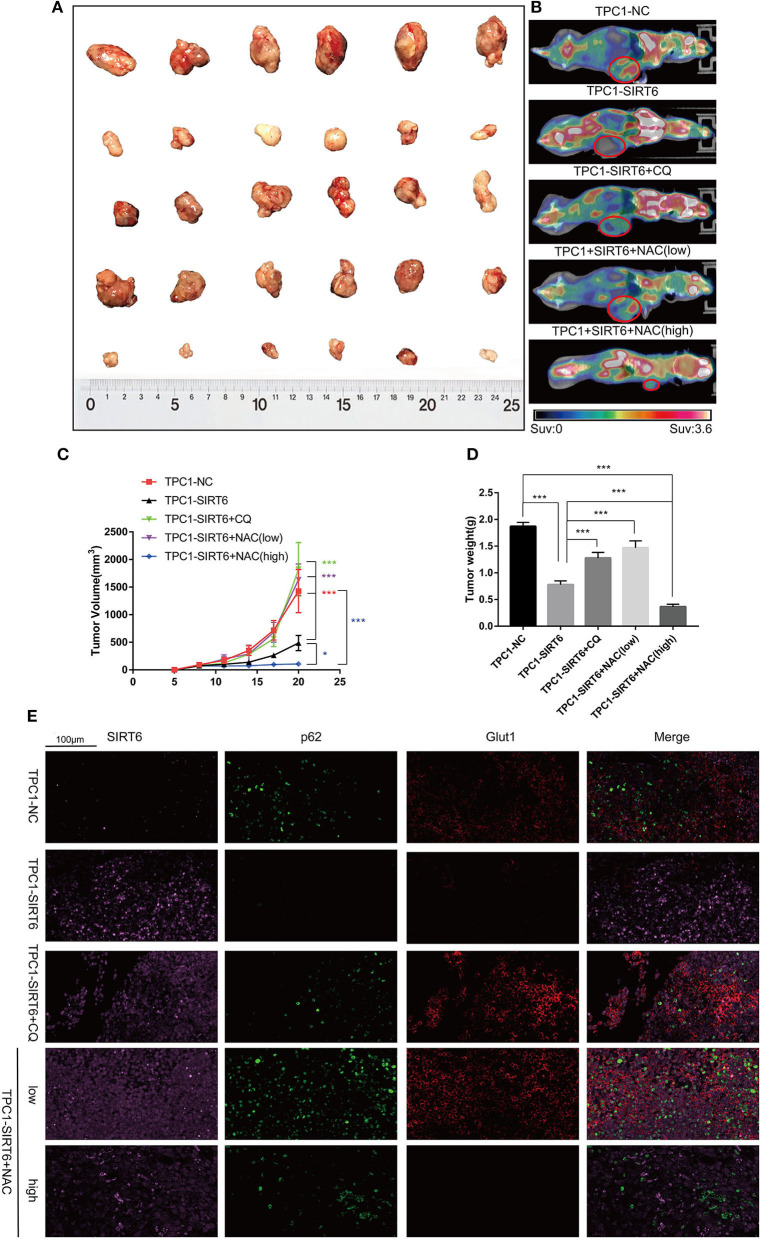
CQ and NAC promoted the growth of SIRT6-overexpressing PTC cells *in vivo*. **(A)** Subcutaneous xenografts derived from each group: TPC1-NC cells, TPC1-SIRT6 cells, TPC1-SIRT6 cells treated with CQ, and TPC1-SIRT6 cells treated with NAC (low: 50 mg/kg, high: 150 mg/kg). **(B)** PET/CT images captured before the sacrifice of mice in each group described above. **(C)** Tumor growth curve of each group described above. **(D)** Tumor weight of each group described above. **(E)** IF staining showing the expression and location of SIRT6, P62, and GLUT1 in each group described above (*p < 0.05 and ***p < 0.001).

A subcutaneous xenograft model was generated, and animals then received different treatments to confirm the results of the *in vitro* experiments *in vivo*. SIRT6 overexpression significantly inhibited tumor growth compared with the NC. Both 50 mg/kg CQ and 50 mg/kg NAC rescued the inhibition of tumor growth in the TPC1-SIRT6 group. However, 150 mg/kg NAC repressed tumor growth compared with the TPC1-SIRT6 and TPC1-NC groups (Figures 5A,C). The changing trend in the tumor weight was similar to the growth curve (Figure 5D). Furthermore, we performed PET/CT before sacrificing the mice to measure the glucose uptake of the xenografts. As expected, the TPC1-SIRT6 group showed a depleted signal (namely, depleted glucose uptake) compared with the TPC1-NC group. Similar to the trend of tumor growth, 50 mg/kg CQ and 50 mg/kg NAC rescued the depletion of glucose uptake, whereas 150 mg/kg NAC aggravated the depletion (Figure 5B). After the mice were sacrificed, the xenografts were subjected to IF staining. As expected, the TPC1-SIRT6 group showed weaker expression of P62 and GLUT1 than the TPC1-NC group. Both CQ and NAC (50 mg/kg) rescued the expression of P62 and GLUT1 in the TPC1-SIRT6 group. In contrast, 150 mg/kg NAC further aggravated the decrease in GLUT1 expression (Figure 5E). The results from the *in vivo* experiment were consistent with the results obtained from the *in vitro* experiments.

### The SIRT-Autophagy-Warburg Effect Axis in Clinical Specimens

**Figure 6:**
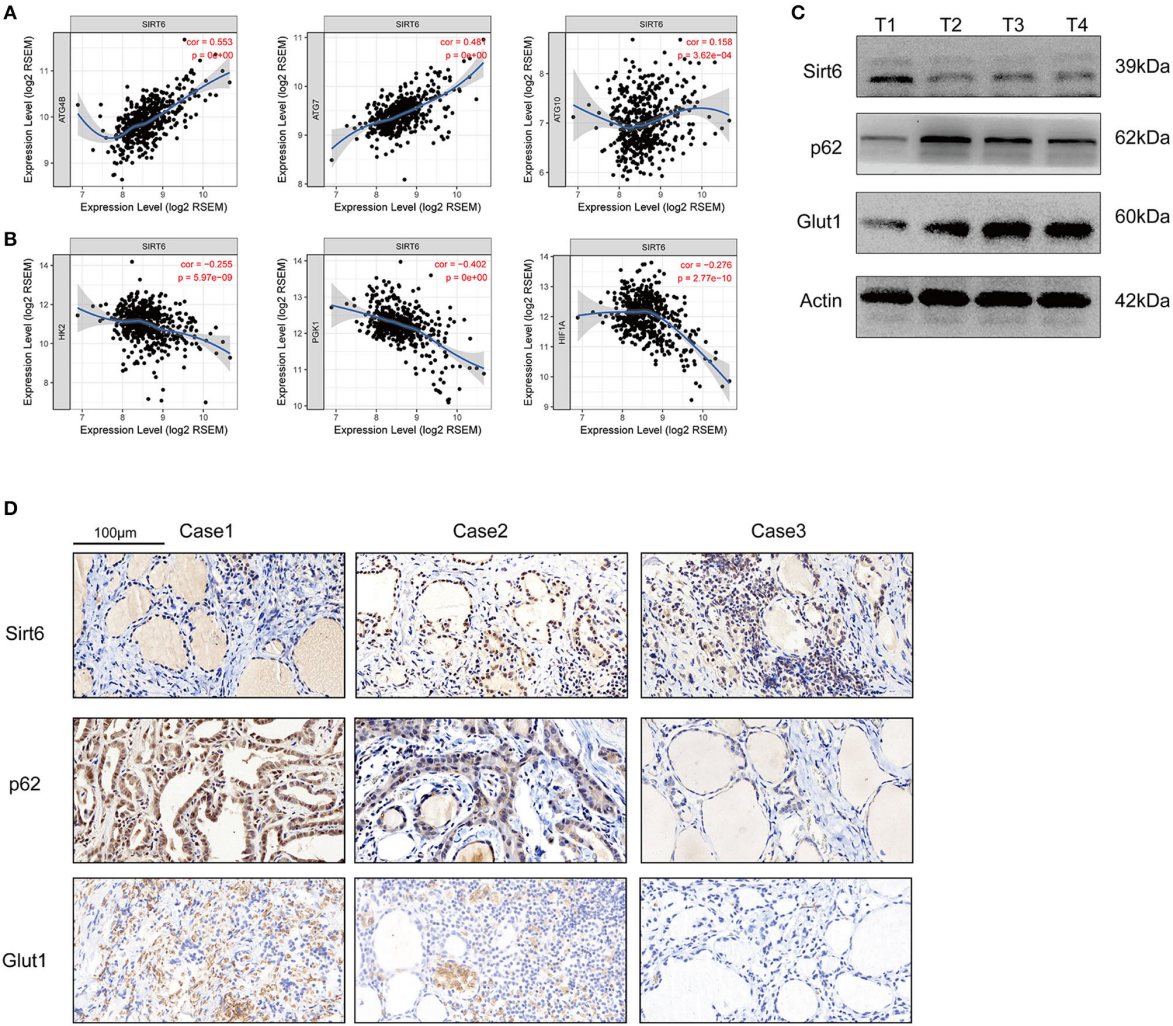
Inspection of the SIRT-autophagy-Warburg effect axis in clinical specimens. **(A)** Co-expression of SIRT6 and autophagy-associated genes (ATGs) analyzed using TIMER web tools based on TCGA database. **(B)** Co-expression of SIRT6 and Warburg effect-associated genes analyzed using TIMER web tools based on TCGA database. **(C)** Western blots showing the levels of the SIRT6, P62, and GLUT1 proteins in PTC specimens. **(D)** IHC staining showing the expression and location of the SIRT6, P62, and GLUT1 proteins in PTC specimens.

Five-hundred-and-one cases in the TCGA database were analyzed using TIMER web tools (https://cistrome.shinyapps.io/timer/) to analyze the SIRT6-autophagy-Warburg effect axis in clinical patients with PTC (21). SIRT6 showed significant co-expression with the autophagy-associated genes ATG4B, ATG7, and ATG10 (Figure 6A), whereas it was not co-expressed with the Warburg effect-associated genes HK2, PGK1, and HIF1A in clinical specimens from patients with PTC (Figure 6B). Subsequently, levels of the SIRT6, P62, and GLUT1 proteins were analyzed in several samples using Western blotting and IHC. SIRT6 was not co-expressed with P62 and GLUT1 (Figures 6C,D).

## Discussion

As shown in our previous study, SIRT6 is upregulated in PTC and promotes tumor invasion and migration by inducing the epithelial-mesenchymal transformation (6). SIRT6 induced both the Warburg effect and autophagy in other diseases, as reported in other published studies. However, in tumors, particularly thyroid tumors, the role of SIRT6 in regulating these two effects remains unclear. Therefore, we determined whether SIRT6 induced the Warburg effect and autophagy in PTC.

We successfully constructed the SIRT6-overexpressing PTC cell lines TPC1-SIRT6/K1-SIRT6 and negative control cell lines TPC1-NC/K1-NC. Subsequently, we performed Western blotting, RT-qPCR, and IF staining for LC3 and TEM of autophagosomes and confirmed that SIRT6 induced autophagy in TPC-1 cells. In a subsequent analysis of the mechanism by which SIRT6 induced autophagy, we speculated that several common signaling pathways and transcription factors play important roles in this interaction, but a negative result was obtained. We then focused on ROS, and the ROS production of TPC1-SIRT6 cells was significantly increased compared with TPC1-NC cells. ER stress is closely related to ROS and autophagy (22). ER stress is characterized by the incorrect folding and aggregation of unfolded proteins in endoplasmic reticulum lumen and a disruption of calcium homeostasis. ER stress activates signaling pathways such as the unfolded protein response and endoplasmic reticulum overload response. PERK, ATF6, and IREL are the three key regulatory pathways involved in ER- stress (23). ROS-related ER stress is strongly correlated with the PERK signaling pathway, as a loss of PERK may lead to defects in cell death sensitivity under pathological conditions that are linked to ROS-mediated ER stress (24). In addition, PERK, eIF2α, ATF4, and CHOP all regulate autophagy (25–27). Therefore, we further detected proteins downstream of the PERK pathway, namely, eIF2α/ATF4/CHOP, and activation was observed in TPC1-SIRT6 cells. TPC1-SIRT6 cells were treated with the ROS inhibitor NAC and ER stress inhibitor 4-PBA to determine the causal link between ROS and ER stress. The ROS inhibitor successfully prevented the activation of eIF2α/ATF4/CHOP signaling, whereas the ER stress inhibitor did not affect ROS levels. Based on this result, the increase in ROS production is upstream of ER stress. Furthermore, both ROS and ER stress inhibitors blocked autophagy in TPC1-SIRT6 cells. All these results confirmed that SIRT6 induced ER stress *via* the PERK pathway, then increased ROS production, and finally induced autophagy.

Thus, far, we have confirmed that SIRT6 induced ER stress by increasing ROS production and subsequently inducing autophagy. However, how SIRT6 increased ROS levels remains unclear. SIRT6 is a class III HDAC that inhibits H3K56ac and H3K9ac (8). We postulated that SIRT6 targeted a ROS regulator at the transcriptional level by regulating H3K56ac or H3K9ac. We investigated the ChIP-sequence dataset GSE102813 that analyzed SIRT6 depletion in BRAF^V600E^ melanoma cells. SIRT6 binds to the NRROS promoter and is accompanied by the H3K56ac peak. Interestingly, BRAF^V600E^ is also the most frequent mutation detected in PTC—similar to melanoma (17). In addition, NRROS is a recently reported negative regulator of ROS, which inhibits ROS production by interacting with NOX2. Coincidentally, NRROS and NOX2 are located in the endoplasmic reticulum and may be involved in ER stress, as described above. By conducting Western blotting, RT-qPCR, and ChIP analyses, we succeeded in verifying our hypothesis. SIRT6 binds to the NRROS promoter and inhibits H3K56ac, thereby inhibiting NRROS expression at the transcriptional level, followed by an increase in ROS production. Accumulated ROS further activate ER stress and finally induce autophagy.

We detected the expression of key genes involved in the Warburg effect to determine the role of SIRT6 in the Warburg effect and found that SIRT6 repressed the expression of these genes at different levels. We then further detected glucose uptake, lactate production, and ATP concentrations in PTC cells. All these parameters decreased significantly in TPC1-SIRT6 cells compared with TPC1-NC cells. Therefore, we confirmed that SIRT6 inhibited the Warburg effect in TPC-1 cells. Many similarities in the Warburg effect and the effect of autophagy on the biological functions of tumors have been reported. In hepatocellular carcinoma, phosphorylation of autophagy-associated gene ATG4B at Ser34 promotes the Warburg effect by inhibiting mitochondrial function and participating in metabolic reorganization (28). In prostate cancer, a strategy targeting both the hexokinase 2-mediated Warburg effect and ULK1-dependent autophagy causes tumor regression in xenografts, leads to near-complete tumor suppression and remarkably prolongs the survival of a PTEN/P53-deficient mouse model (29). In A549 alveolar adenocarcinoma cells, the downregulation of the PKM2 gene involved in the Warburg effect gene induced apoptosis and autophagy. and this autophagy protected the cells from apoptotic cell death (30). In summary, the Warburg effect and autophagy are closely related to the occurrence and development of tumors, and their regulatory networks overlap to some extent. However, few studies have examined the relationship between the Warburg effect and autophagy in thyroid cancer.

TPC1-SIRT6 cells were treated with NAC (5 mM) and the autophagy inhibitor CQ to detect indicators of the Warburg effect and to further study the interaction of the Warburg effect and autophagy. Both NAC and CQ rescued the inhibition of the Warburg effect in TPC1-SIRT6 cells. Based on this result, SIRT6 induced autophagy by increasing ROS production, subsequently inhibiting the Warburg effect. As the autophagy-lysosome pathway is the main mechanism for protein degradation, we suggested that SIRT6 decreased the levels of proteins involved in the Warburg effect by inducing the autophagic degradation of GLUT1. As expected, the lysosome inhibitor CQ, but not the proteasome inhibitor MG-132, rescued the expression of GLUT1. Furthermore, SIRT6 depleted GLUT1 in the membrane and promoted its co-localization with autophagosome marker LC3. Therefore, we inferred that SIRT6 inhibited the Warburg effect by inducing the autophagic degradation of GLUT1.

Our results contradict the findings of previous studies. ROS are a class of oxygen atoms or clusters formed by oxygen that induce the Warburg effect by activating the AMPK pathway (31–33). In the present study, increased ROS levels were accompanied by the inhibition of the Warburg effect. Therefore, we further increased the concentration of NAC used to treat TPC1-SIRT6 cells. Interestingly, 15 mM NAC further inhibited autophagy, but restrained the Warburg effect. At 5 mM, NAC did not alter the activation of the AMPK pathway, but at 10–15 mM, NAC significantly inhibited the AMPK pathway. After TPC1-SIRT6+NAC (15 mM) cells were treated with the AMPK activator, the indexes of the Warburg effect were successfully rescued. Furthermore, AMPK activation directly increased autophagy. Apparently, ROS-induced autophagy and AMPK pathway both participated in regulating the Warburg effect. Previous studies have also reported the interaction of ROS and AMPK. SIRT6 activated the AMPK pathway by regulating H3K9ac and H3K56ac in podocytes, thus preventing ROS-induced mitochondrial dysfunction and apoptosis (34). Furthermore, SIRT6 promoted autophagy through the ROS-mediated activation of the AMPK pathway in cancer cell lines (35). In the present study, ROS inhibition showed a stronger association with the expression Warburg effect-related genes than AMPK activation. Therefore, we considered ROS as an initial regulator of both autophagy and the AMPK pathway in the mechanism regulating the Warburg effect. We propose that the balance between the activation of the AMPK pathway and autophagy is crucial for the ROS-mediated regulation of the Warburg effect. Weaker inhibition of ROS may induce the Warburg effect by repressing autophagy. However, stronger inhibition of ROS conquers autophagy and represses the Warburg effect by activating the AMPK pathway.

Although our previous study showed that SIRT6 promoted the invasion of PTC *via* the EMT, SIRT6 also repressed the Warburg effect in the present study. The role of SIRT6 in PTC appears to be more complex and it has other roles than an oncogene. The Warburg effect is an important basis for the rapid growth of tumors, as observed using a PET/CT examination. The uptake rate and concentration of contrast media (such as 18F-FDG) are higher in tumors with an active Warburg effect (36). Therefore, we determined the effect of the SIRT6-ER stress-autophagy-Warburg effect axis on the growth of xenografts in an animal model using 18F-FDG PET/CT. SIRT6 overexpression significantly inhibited the Warburg effect and tumor growth; however, CQ rescued the inhibition. CQ is a drug that treats cancer by inhibiting autophagy or other mechanisms (37, 38). In the present study, CQ promoted tumor growth by increasing the Warburg effect in SIRT6-overexpressing PTC cells. Therefore, CQ should be more cautiously applied in patients as a treatment for PTC and SIRT6 expression must be assessed. Similarly, NAC plays a dual role in the Warburg effect and growth of tumors. Notably, 50 mg/kg NAC significantly increased the Warburg effect and tumor growth, whereas 150 mg/kg NAC restrained the Warburg effect and tumor growth instead. In previous studies, NAC has also been considered to be an anticancer drug that inhibits the growth of tumors by blocking oxidative stress and other mechanisms (39, 40). However, a different result was observed in TPC1-SIRT6 cells. Therefore, a higher concentration of NAC should be administered to patients with PTC overexpressing SIRT6.

Our study has several limitations. First, we performed numerous experiments to verify the SIRT6 -ROS-autophagy-Warburg effect regulatory axis *in vitro*. Although we used a large sample from the TCGA database to compensate for the deficiency of this study to some extent, the results were not verified in a large number of human samples. Second, in this paper, we mainly studied the regulatory effect of SIRT6 on the Warburg effect through changes in autophagy. HIF1-α is an important factor regulating the Warburg effect. However, it was not the focus of our study. We will consider evaluating this regulatory mechanism in subsequent studies. Moreover, in a previous publication, we showed that SIRT6 was associated with more aggressive characteristics, whereas here, we suggest that SIRT6 overexpression inhibits tumorigenesis. Notably, the effects of a single gene (such as P53 and TGF-β) on tumor progression are complex and dynamic. The findings from the present study, that SIRT6 inhibits the Warburg effect, are a good supplement to the results from our previous work.

## Conclusions

**Figure 7:**
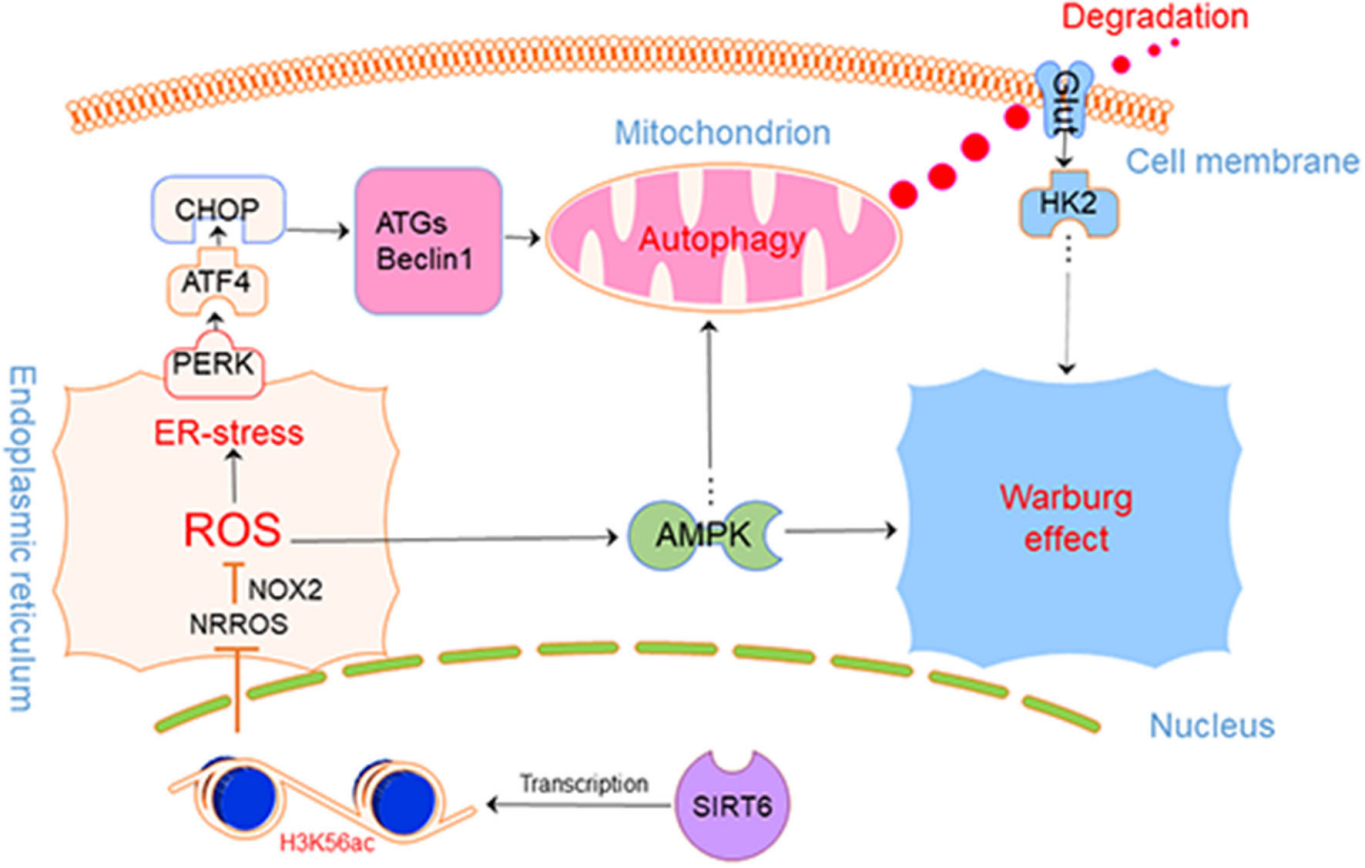
SIRT6-Autophagy-Warburg effect axis in PTC.

Based on our findings, we safely draw the conclusion that SIRT6 promotes autophagy in PTC cells by increasing ROS production and upregulating autophagy to further inhibit the Warburg effect, by inducing the degradation of GLUT1. Furthermore, the AMPK pathway provides negative feedback inhibition of this process (Figure 7).

## Data Availability Statement

The original contributions presented in the study are included in the article/Supplementary Material, further inquiries can be directed to the corresponding author/s.

## Ethics Statement

The animal study was reviewed and approved by the Institutional Animal Care and Utilization Committee of Fudan University Pudong Animal Experimental Center. This study was approved by the Ethical Committee of Shanghai Pudong Hospital. All patients provided their written informed consent to participate. Written informed consent for publication was obtained from all participants.

## Author Contributions

ZY, RH, and XW contributed to performing the experiments and analyzing the data. WY contributed to specimen collection and animal models. ZM and MY contributed to the design of the study. All authors read and approved the final version of the manuscript.

## Conflict of Interest

The authors declare that the research was conducted in the absence of any commercial or financial relationships that could be construed as a potential conflict of interest.

## Supplementary Material

Figure S1 **(A)** Levels of proteins involved in the PERK/ATF4/CHOP pathway in K1 cells from each group. **(B)** Levels of autophagy-associated proteins in K1 cells from each group. **(C)** Levels of proteins associated with the Warburg effect in K1 cells from each group. **(D)** ROS production in each group of K1 cells detected using DCFH-DA. **(E)** Glucose uptake of each group of K1 cells. **(F)** Lactate production in K1 cells from each group. **(G)** ATP content in each group of K1 cells. **(H)** Autophagosomes were monitored using IF staining for LC3 in K1 cells. **(I)** Images of IF staining for P62 in TPC1-SIRT6 and TPC1-NC cells (All compared with K1-SIRT6, ^*^*p* < 0.05, ^**^*p* < 0.01, and ^***^*p* < 0.001).

Figure S2 **(A)** Expression of GLUT1 at different time points 24 h after the withdrawal of the 24-h CHX treatment normalized to Actin (^*^k1-NC vs. k1-SIRT6, ^*^*p* < 0.05; ^#^k1-SIRT6 vs. k1-SIRT6+CQ, ^#^*p* < 0.05). **(B)** IF staining showing the expression and location of GLUT1 and LC3 in K1 cells. **(C)** ECAR in each group of K1 cells detected using the Seahorse XF 96 analyzer. **(D)** OCR in each group of K1 cells detected using the Seahorse XF 96 analyzer (^*^*p* < 0.05 and ^***^*p* < 0.001).

## Abbreviations

ATGs: autophagy-associated genes
CQ: chloroquine
EMT: epithelial-mesenchymal transition
ER stress: endoplasmic reticulum stress
Glut1: glucose transporter 1
H3K56ac: histone H3 lysine 56 acetylation
NAC: N-acetyl-L-cysteine
NRROS: negative regulator of reactive oxygen species
PTC: papillary thyroid cancer.
