# Supplementary figures and images for: The SIRT6-Autophagy-Warburg Effect Axis in Papillary Thyroid Cancer
Source: Front Oncol. 2020 Aug 28;10:1265. doi: 10.3389/fonc.2020.01265 (PMC7485319; doi:10.3389/fonc.2020.01265)

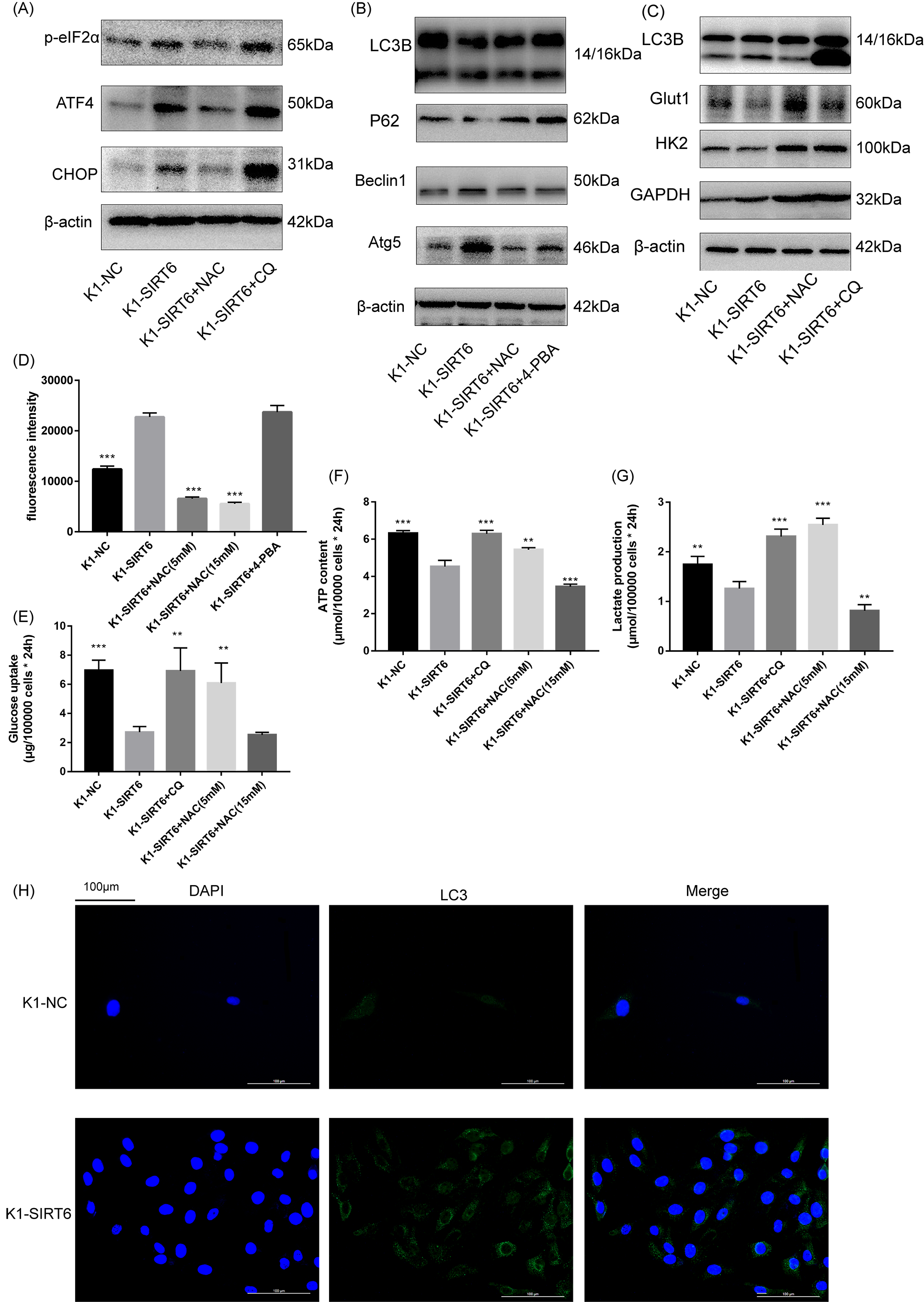

Supplement: Figure S1 — (A) Levels of proteins involved in the PERK/ATF4/CHOP pathway in K1 cells from each group. (B) Levels of autophagy-associated proteins in K1 cells from each group. (C) Levels of proteins associated with the Warburg effect in K1 cells from each group. (D) ROS production in each group of K1 cells detected using DCFH-DA. (E) Glucose uptake of each group of K1 cells. (F) Lactate production in K1 cells from each group. (G) ATP content in each group of K1 cells. (H) Autophagosomes were monitored using IF staining for LC3 in K1 cells. (I) Images of IF staining for P62 in TPC1-SIRT6 and TPC1-NC cells (All compared with K1-SIRT6, *p < 0.05, **p < 0.01, and ***p < 0.001). [file Image_1.TIF]

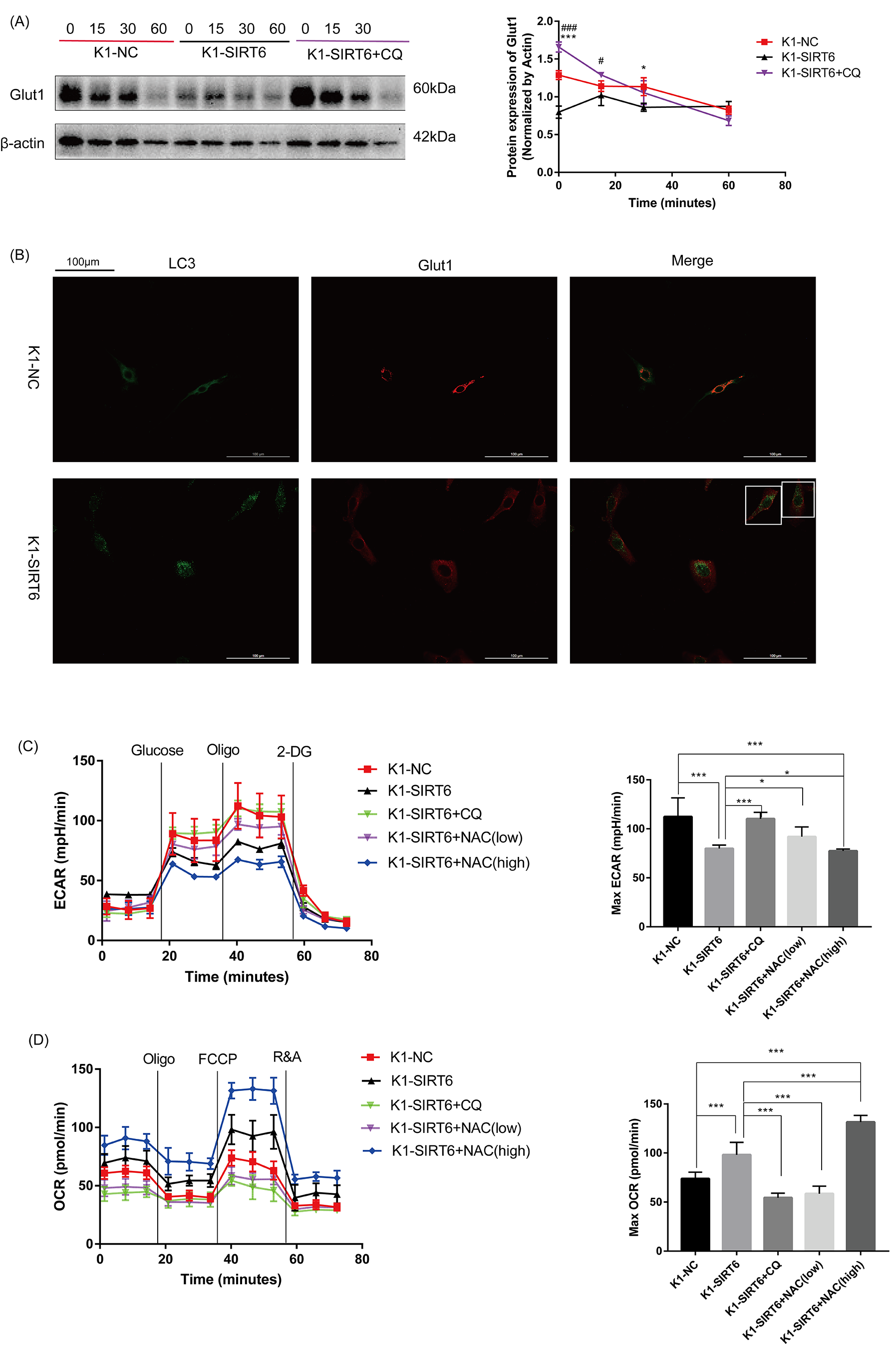

Supplement: Figure S2 — (A) Expression of GLUT1 at different time points 24 h after the withdrawal of the 24-h CHX treatment normalized to Actin (*k1-NC vs. k1-SIRT6, *p < 0.05; #k1-SIRT6 vs. k1-SIRT6+CQ, #p < 0.05). (B) IF staining showing the expression and location of GLUT1 and LC3 in K1 cells. (C) ECAR in each group of K1 cells detected using the Seahorse XF 96 analyzer. (D) OCR in each group of K1 cells detected using the Seahorse XF 96 analyzer (*p < 0.05 and ***p < 0.001). [file Image_2.TIF]
